# Serum albumin levels as a predictive biomarker for low-load resistance training programs’ effects on muscle thickness in the community-dwelling elderly Japanese population: interventional study result

**DOI:** 10.1186/s12877-021-02403-7

**Published:** 2021-08-18

**Authors:** Shuji Sawada, Hayao Ozaki, Toshiharu Natsume, Daiki Nakano, Pengyu Deng, Toshinori Yoshihara, Takuya Osawa, Hiroyuki Kobayashi, Shuichi Machida, Hisashi Naito

**Affiliations:** 1 COI Project Center, Juntendo University, 2-1-1 Hongo, Bunkyo-ku, Tokyo 113-8421 Japan; 2 School of Health and Sports Science, Juntendo University, 1-1 Hirakagakuendai, Inzai, Chiba 270-1695 Japan; 3 School of Sport and Health Science, Tokai Gakuen University, 21-233 Nishinohora, Ukigai, Miyoshi, Aichi 470-0207 Japan; 4 Department of Human Structure & Function, Tokai University School of Medicine, 143 Shimokasuya, Isehara, Kanagawa 259-1193 Japan; 5 Ritsumeikan Global Innovation Research Organization, Ritsumeikan University, 1-1-1 Noji-higashi, Kusatsu, Shiga 525-8577 Japan; 6 Graduate School of Health and Sports Science, Juntendo University, 1-1 Hirakagakuendai, Inzai, Chiba 270-1695 Japan; 7 Faculty of Sports and Health Sciences, Japan Women’s College of Physical Education, 8-19-1, Kitakarasuyama, Setagaya-ku, Tokyo 157-8565 Japan; 8 Department of Internal Medicine, Mito Kyodo General Hospital, University of Tsukuba, 3-2-7 Miyamachi, Mito, Ibaraki 310-0015 Japan; 9 Institute of Health and Sports Science & Medicine, Juntendo University, 1-1 Hirakagakuendai, Inzai, Chiba 270-1695 Japan

**Keywords:** Aged, Resistance training, Serum albumin, Muscle hypertrophy

## Abstract

**Background:**

Resistance training has been recommended as an effective measure against age-related loss of muscle mass and muscle strength, called sarcopenia, even in older adults. However, despite subjecting each participant to the same training program, the training effect solely depended on the individual. This study aimed to evaluate whether certain blood parameters influenced the effect of a low-load resistance training program on muscle thickness in the community-dwelling elderly population.

**Methods:**

Sixty-nine community-dwelling Japanese (49 women and 20 men) subjects aged 69.4 ± 6.5 years were included. Low-load resistance training was performed twice a week for 12 weeks. Muscle thickness at the anterior aspects of the thigh (AT) was measured using a B-mode ultrasound device, and 22 blood parameter levels were assessed before and after the program. We checked the first quartile value of each parameter to establish cutoff values, and participants were divided into low or normal groups for each parameter.

**Results:**

A low-load resistance training program significantly increased muscle thickness at the AT. The interaction between time and groups was examined at low (< 4.1 g/dL) versus normal (≥ 4.1 g/dL) serum albumin (Alb) levels. Although there was no difference in muscle thickness at the AT before the training intervention, the hypertrophic effects were higher in the normal serum Alb level group than in the low serum Alb level group. The binomial logistic regression analysis showed that participants in the low serum Alb group had an odds ratio of 7.08 for decreased muscle thickness at the AT. The effect of a low-load resistance training program on lower limb muscle thickness appears to be limited in participants with low serum Alb levels before training interventions.

**Conclusions:**

Serum Alb level may act as a biomarker to predict the effects of low-load resistance training programs on muscle hypertrophy in elderly individuals.

**Trial registration:**

This study was retrospectively registered in UMIN-Clinical Trial Registry (CTR), ID: UMIN000042759 (date of registration, 14 Dec 2020).

## Background

Skeletal muscle plays an important role in physical functions related to the activities of daily living [1] and in systemic energy regulation controlled by glucose and lipid metabolism [2]. Despite playing a major role in systemic homeostasis, skeletal muscle size and strength both decrease with age [3, 4]. This age-related loss of muscle mass and muscle strength, called sarcopenia, is related to a wide range of chronic disorders [5–8], motor disorders [9, 10], and mortality [11]. Because of this, sarcopenia is a primary therapeutic target for improving the effects of aging in the elderly, and it has been reported that exercise intervention, especially involving resistance training, is a key approach for preventing and improving sarcopenia [12, 13].

Resistance training has been recommended as an effective method against the problems indicated above, even in older adults [14, 15]. Although high-load resistance training is the primary method to reduce the effects of aging, this type of training generally requires high external loads, which may be difficult for older adults to conduct safely. To identify an appropriate approach for older adults, we found that a low-load resistance training program could induce muscle hypertrophy in middle-aged and older adults [16]. However, despite subjecting each participant to the same training program, the training effect solely depended on the individual. In a previous study, it was reported that calorie restriction accelerated the catabolism of the lean body mass [17], and the effect of resistance exercise was significantly greater in the exercise and nutrition intervention group than in the exercise only group [18]. Therefore, the participants with malnutrition could only show limited training effect, and we hypothesized that we could assess this risk using blood parameters.

It is well known that blood parameters are effective screening tools for identifying the risk of metabolic syndromes, locomotive syndromes, and other multi-factorial diseases [19–21]. To diagnose diseases early, annual medical checkups that include complete blood counts and blood biochemistry panels have been conducted in Japan. The consultation rate of annual medical checkups was reported to be 63.7 % [22], which indicates the percentage of Japanese citizens managing their health through annual checkups. From this viewpoint, we focused on blood parameters, including complete blood counts and blood biochemistry panels, and hypothesized that these blood parameters might affect skeletal muscle adaptation in response to a low-load resistance training program. To test this, we first comprehensively verified the blood parameters, including complete blood count and blood biochemistry, which entailed annual medical checkups conducted in Japan, before and after a low-load resistance training program. Should an evidence of a relationship between the status of these blood parameters and the effect of a low-load resistance training program be revealed, several parameters could then be used as biomarkers to predict the effects of low-load resistance training programs on muscle hypertrophy in elderly individuals.

This study aimed to evaluate whether the levels of some blood parameters influenced the effect of a low-load resistance training program on lower limb muscle thickness in community-dwelling middle-aged and elderly individuals.

## Methods

### Participants

Community-dwelling middle-aged and elderly Japanese were recruited to participate in this study through printed advertisements in a public information magazine. They were informed about the methods, procedures, and risks and provided written informed consent before participating. We excluded individuals who did not follow our instructions or those with medical conditions that could limit their ability to participate in the resistance training program, based on the decision of the physician in charge. Sixty-nine participants aged 69.4 ± 6.5 years (49 women and 20 men) volunteered for this study. Although 19, 16, 6, and 4 participants had a medical history of hypertension, dyslipidemia, heart failure, and diabetes mellitus, respectively, and accordingly, some of them were using medications including atorvastatin and amlodipine, they were all approved to participate in the exercise program by the physician in charge. Their heights, weights, muscle thicknesses, and blood parameters were evaluated before (pre) and after (post) the training period. This study was conducted in accordance with the Declaration of Helsinki and was approved by the Ethics Committee for Human Experiments of Juntendo University, Chiba, Japan (Approval Number: 27–52). This study was registered in UMIN-Clinical Trial Registry (CTR) (ID: UMIN000042759, Date of registration: December 14, 2020).

### Training program

Participants were instructed to engage in a low-load resistance training program using their own body weight and an elastic band. They were also instructed to avoid changing their dietary patterns throughout the training period. The program was conducted twice a week for 12 weeks, and the total number of classes was 22 or 23. The program was composed of nine exercises: squats, split squats, push-ups, heel raises, crunches, hip lifts, seated rows, shoulder presses, and arm curls. The first 6 exercises involved the participant’s own body weight, and the last three exercises required an elastic band (Thera-Band®; The Hygenic Corporation, Akron, OH, USA). In the first 2 weeks of the training period, the program was composed of only 4 exercises: squats, push-ups, crunches, and hip lifts, and the participants performed 3 sets of 8 repetitions with a 60-s rest between each set. In each repetition, they were instructed to spend 3 s in both the concentric and eccentric phases. After the first 2 weeks, the number of exercises per session, repetitions, sets per exercise, and exercise times were gradually increased, and the rest interval was gradually decreased every 2 weeks throughout the 12-week training period. For each session, it was indicated to the participants to continue until muscle fatigue. This training program was conducted according to the protocol followed in a previous study, and the details are described by Ozaki et al. [16].

### Muscle thickness

Each participant’s muscle thickness was measured with a B-mode ultrasound device using a 5 to 18-MHz scanning head (Noblus; Aloka, Tokyo, Japan). We evaluated the anterior aspects of the thigh (AT) at the midpoint between the greater trochanter and lateral condyle of the femur. The participants were required to rest in the sitting position for at least 30 min before the measurement and to be in a supine position during the measurement. All the measurements were performed by the same operator. Before the study, we conducted preliminary examination to check the reproducibility and calculated the test–retest (inter-session) reliability using intraclass correlation coefficient (ICC), standard errors of measurement (SEM), and minimal difference. The ICC, SEM, and minimal difference for the muscle thickness in the AT was determined in 10 older men and women as follows: 0.992, 0.37 mm, 1.03 mm, respectively. This measurement was also conducted according to the method used in a previous study, and the details are described by Ozaki et al. [16].

### Blood parameters

Venous blood samples of approximately 13 mL were obtained following at least 2 h of fasting before (pre) and after (post) the 12-week training program, and the levels of the following blood parameters were assessed: white blood cell count (WBC), red blood cell count (RBC), hemoglobin (Hb), hematocrit (Ht), mean corpuscular volume (MCV), mean corpuscular hemoglobin (MCH), mean corpuscular hemoglobin concentration (MCHC), platelet count (PLT), total protein (TP), albumin (Alb), aspartate aminotransferase (glutamic oxaloacetic transaminase; AST [GOT]), alanine aminotransferase (glutamic pyruvic transaminase; ALT [GPT]), alkaline phosphatase (ALP), leucine aminopeptidase (LAP), lactate dehydrogenase (LD [LDH]), γ-glutamyl transferase (γ-GTP), triglycerides (TG), total cholesterol (TC), high-density lipoprotein cholesterol (HDL-C), low-density lipoprotein cholesterol (LDL-C), hemoglobin A1c (HbA1c), and fasting blood sugar (FBS). These items were monitored using the complete blood count and blood biochemistry tests, which are included in annual medical checkups conducted in Japan.

We checked each blood parameter’s first quartile value before (pre) the training program, to establish the following cutoff criteria for the reduced levels: WBC = 4400/µL, RBC = 414 × 10^4^/µL, Hb = 12.6 g/dL, Ht = 37.9 %, MCV = 87.7 fL, MCH = 29.2 pg, MCHC = 32.8 %, PLT = 18.8 × 10^4^/µL, TP = 7 g/dL, Alb = 4.1 g/dL, AST(GOT) = 18 U/L, ALT(GPT) = 14 U/L, ALP = 178 U/L, LAP = 45 U/L, LD(LDH) = 169 U/L, γ-GTP = 14 U/L, TC = 181 mg/dL, HDL-C = 52 mg/dL, TG = 85 mg/dL, LDL-C = 102 mg/dL, HbA1c = 5.3 %, and FBS = 95 mg/dL. Using criteria for each blood parameter, participants were divided into the first quartile group, which is the lowest quartile group, as participants in this group have relatively low blood parameter values, or into the combined second, third, and fourth quartile groups with participants having normal blood parameter values.

### Statistical analyses

To verify the training effect on muscle thickness at the AT, we analyzed the parameters before (pre) and after (post) the training program. Data were analyzed using the paired t-tests, two-way analysis of variance, analysis of covariance, and binomial logistic regression analysis. When there was a significant interaction in the two-way analysis of variance between time and group, the simple main effects analysis was conducted. In the binomial logistic regression analysis, we included age and sex as confounding factors, and the variable selection was conducted using a stepwise method with *p* = 0.20. *P* less than 0.05 was considered statistically significant. Results were expressed as means and standard deviations, and the odds ratio was represented by means and 95 % confidence intervals. Statistical analyses were performed using BellCurve for Excel (Social Survey Research Information Co., Ltd., Japan).

## Results

The participants’ average height was 156.8 ± 7.6 cm, and the average weight was 59.1 ± 10.3 kg. The average participation rate of the classes was 87.4 % (range: 63.6–100.0 %). Of the 69 participants, 4 had smoking habit, 12 drank alcohol every day, and 24 drank alcohol sometimes.

**Fig. 1:**
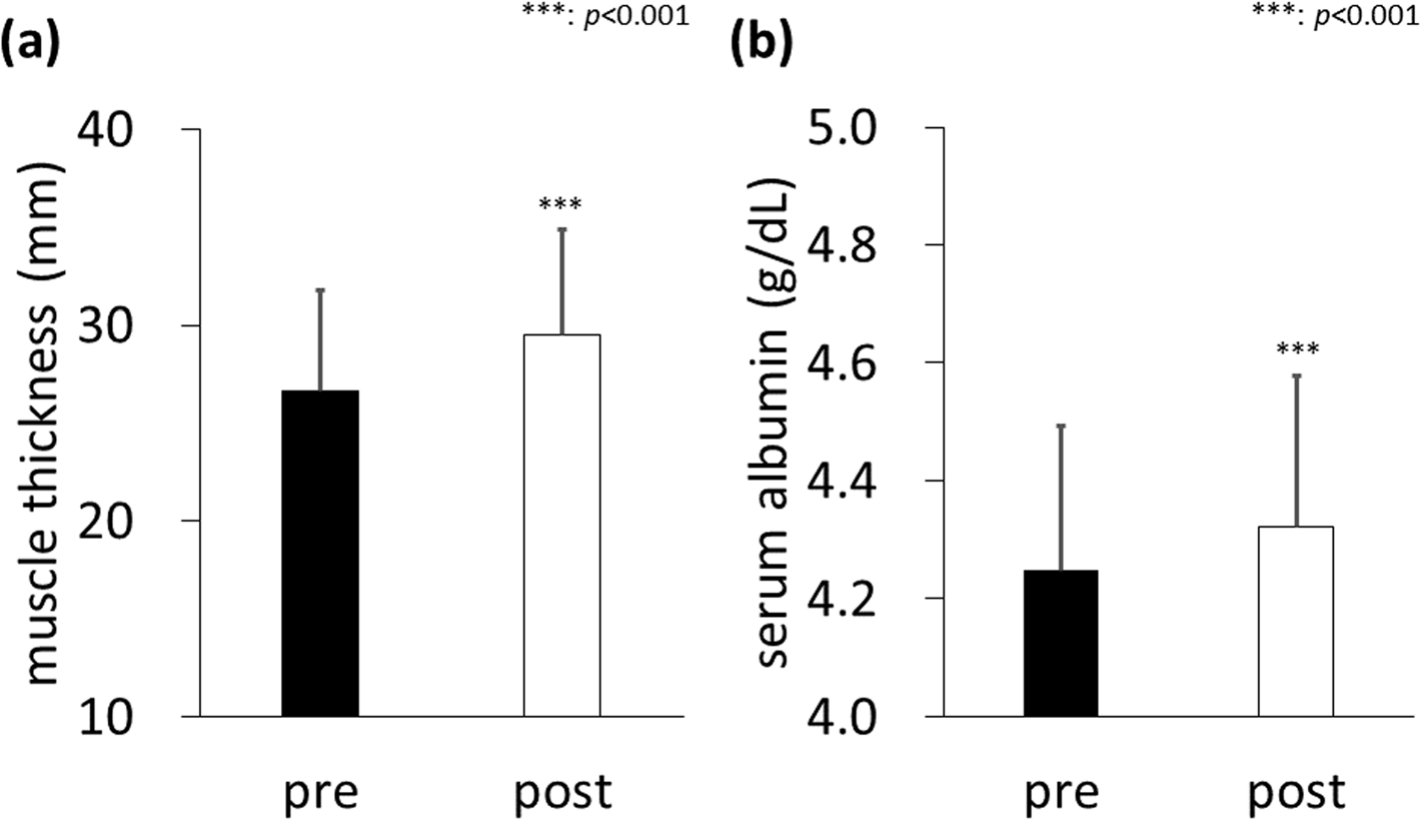
Effect of a low-load resistance training program on (**a**) muscle thickness, and (**b**) serum albumin levels. Data are presented as mean ± standard deviation (SD). *N* = 69

The average muscle thickness at the AT of all participants was 26.65 ± 5.12 mm before the training intervention, which significantly increased to 29.48 ± 5.38 mm after undergoing the biweekly low-load resistance training program for 12 weeks (*p* < 0.001, Fig. 1a). The average serum Alb concentration of all participants was 4.25 ± 0.25 g/dL before the training intervention, which significantly increased to 4.32 ± 0.26 g/dL after the training program (*p* < 0.001, Fig. 1b).

**Fig. 2:**
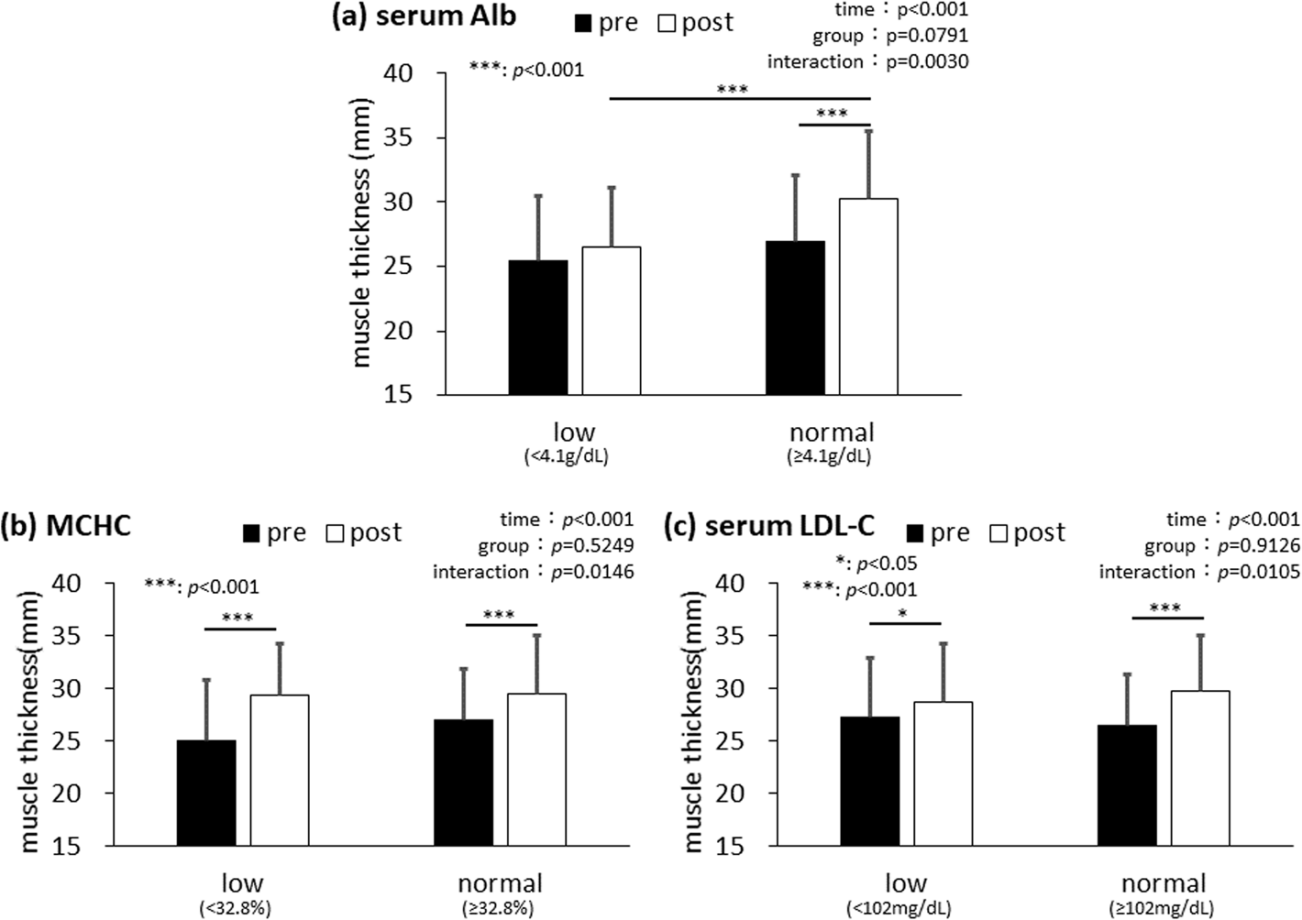
Comparison of changes in muscle thickness for (**a**) low and normal serum albumin (Alb) levels, **b** low and normal mean corpuscular hemoglobin concentration (MCHC), and **c** low serum and normal low-density lipoprotein-cholesterol (LDL-C) levels. Data were analyzed using two-way analysis of variance; the main effects on time, but not on group, and the time × group interactions were significant for all three measurements. Although the time × group interactions were detected in all three measurements, a significant difference after training intervention was only detected in serum Alb levels compared with the low and normal groups using Tukey’s multiple comparison test as a post-hoc analysis. Data are presented as mean ± standard deviation (SD). *N* = 69

Using the blood parameter criteria, we checked the first quartile value of each blood parameter to establish the cutoff criteria for reduced levels (Table 1). When the two-way analysis of variance was conducted, the effects were significant for time, but not for group, and the time × group interactions were significant for low (< 32.8 %) versus normal (≥ 32.8 %) MCHC, low (< 4.1 g/dL) versus normal (≥ 4.1 g/dL) serum Alb levels, and low (< 102 mg/dL) versus normal (≥ 102 mg/dL) serum LDL-C levels (*p* < 0.05; Table 2). When comparing the low and normal serum Alb level groups using the simple main effects analysis, muscle thickness at the AT was only significantly increased in the normal serum Alb level group (pre: 26.96 ± 5.13 mm, post: 30.26 ± 5.31 mm, *p* < 0.001; Fig. 2a). Additionally, there was no difference in muscle thickness at the AT before the training intervention; however, participants in the normal serum Alb level group had greater muscle thickness after the training intervention than those in the low serum Alb level group (low: 26.45 ± 4.68 mm, normal: 30.26 ± 5.31 mm, *p* < 0.001; Fig. 2a). To verify the effect of participation rate of the classes on serum Alb level and muscle thickness at the AT, an analysis of covariance was conducted. Although regression parallelism was confirmed, the regression was not significant. Time × group interactions were also detected in the MCHC and serum LDL-C levels (*p* < 0.001; Table 2). When comparing the low and normal MCHC groups using the simple main effects analysis, muscle thickness at the AT were significantly increased both in the low MCHC group (pre: 25.05 ± 5.82 mm, post: 29.45 ± 4.83 mm, *p* < 0.001; Fig. 2b) and the normal MCHC group (pre: 27.02 ± 4.93 mm, post: 29.49 ± 5.54 mm, *p* < 0.001; Fig. 2b); however, the increase was greater in the low MCHC group. On the other hand, when comparing the low and normal MCHC groups using the simple main effects analysis, muscle thickness at the AT was significantly increased both in the low serum LDL-C level group (pre: 27.22 ± 5.77 mm, post: 28.67 ± 5.63 mm, *p* < 0.05; Fig. 2c) and the normal serum LDL-C level group (pre: 26.46 ± 4.94 mm, post: 29.75 ± 5.32 mm, *p* < 0.001; Fig. 2c); however, the increase was greater in the normal serum LDL-C level group.

**Table 1:** The first quartile value of each blood parameter

	Unit	Mean Value	Minimum Value	Maximum Value	Median Value	1^st^ Quartile Value	Interquartile Range
WBC	/μL	5369.6	2800	8400	5300	4400	1900
RBC	×10^4^/μL	439.7	366	552	435	414	49
Hb	g/dL	13.2	10.3	16.2	13.2	12.6	1.2
Ht	%	39.64	32.8	48	39.6	37.9	3.3
MCV	fL	90.35	77.5	101	89.9	87.7	5.5
MCH	pg	30.09	25.5	33.5	30.1	29.2	1.9
MCHC	%	33.3	30.9	34.7	33.2	32.8	1.0
PLT	×10^4^/μL	22.78	13.2	47.1	21.5	18.8	6.7
TP	g/dL	7.23	6.6	8	7.2	7	0.5
Alb	g/dL	4.25	3.7	4.9	4.2	4.1	0.3
AST(GOT)	U/L	21.6	12	41	21	18	5
ALT(GPT)	U/L	19.4	7	63	16	14	7
ALP	U/L	216.7	74	387	211	178	68
LAP	U/L	48.2	32	83	47	45	5
LD(LDH)	U/L	190.9	135	276	189	169	43
γ-GTP	U/L	23.4	10	107	20	14	13
TG	mg/dL	140.7	46	393	132	85	86
TC	mg/dL	209.7	148	307	209	181	48
HDL-C	mg/dL	62.2	29	101	60	52	20
LDL-C	mg/dL	121.2	64	211	114	102	36
HbA1c	%	5.57	4.7	7.3	5.5	5.3	0.4
FBS	mg/dL	106.6	72	191	102	95	16

*WBC* white blood cell count, *RBC* red blood cell count, *Hb* hemoglobin, *Ht* hematocrit, *MCV* mean corpuscular volume, *MCH* mean corpuscular hemoglobin, *MCHC *mean corpuscular hemoglobin concentration, *PLT* platelet count, *TP* total protein, *Alb* albumin, *AST(GOT)* aspartate aminotransferase (glutamic oxaloacetic transaminase), *ALT(GPT)* alanine aminotransferase (glutamic pyruvic transaminase), *ALP* alkaline phosphatase, *LAP* leucine aminopeptidase, *LD(LDH)* lactate dehydrogenase, *γ-GTP* γ-glutamyltransferase, *TG *triglyceride, *TC* total cholesterol, *HDL-C* high-density lipoprotein cholesterol, *LDL-C* low-density lipoprotein cholesterol, *HbA1c* hemoglobin A1c, *FBS* fasting blood sugar

**Table 2:** Summarized results of the two-way analysis of variance

	n(female:male)		muscle thickness(mm)				time	group	interaction
	pre		pre		post				
	low	normal	low	normal	low	normal	*p* value		
WBC	17(15:2)	52(34:18)	23.9 ± 4.6	27.6 ± 5.0	27.7 ± 5.3	30.1 ± 5.3	**< 0.001**	**0.0306**	0.082
RBC	17(14:3)	52(35:17)	24.5 ± 4.6	27.3 ± 5.1	27.4 ± 5.2	30.2 ± 5.3	**< 0.001**	**0.0461**	0.9887
Hb	16(15:1)	53(34:19)	23.8 ± 3.1	27.5 ± 5.3	26.8 ± 3.9	30.3 ± 5.5	**< 0.001**	**0.0118**	0.8063
Ht	17(15:2)	52(34:18)	25.2 ± 4.4	27.1 ± 5.3	27.7 ± 5.2	30.1 ± 5.4	**< 0.001**	0.1295	0.5764
MCV	17(12:5)	52(37:15)	26.7 ± 5.0	26.6 ± 5.2	29.1 ± 4.7	29.6 ± 5.6	**< 0.001**	0.8868	0.5129
MCH	17(14:3)	52(35:17)	25.2 ± 4.6	27.1 ± 5.2	28.7 ± 4.8	29.8 ± 5.6	**< 0.001**	0.297	0.2858
MCHC	13(12:1)	56(37:19)	25.0 ± 5.8	27.0 ± 4.9	29.5 ± 4.8	29.5 ± 5.5	**< 0.001**	0.5249	**0.0146**
PLT	17(9:8)	52(40:12)	27.1 ± 5.1	26.5 ± 5.2	29.5 ± 4.4	29.5 ± 5.7	**< 0.001**	0.8204	0.4596
TP	17(11:6)	52(38:14)	23.4 ± 3.6	27.7 ± 5.1	26.9 ± 4.9	30.3 ± 5.3	**< 0.001**	**0.0057**	0.2622
Alb	14(9:5)	55(40:15)	25.4 ± 5.1	27.0 ± 5.1	26.5 ± 4.7	30.3 ± 5.3	**< 0.001**	0.0791	**0.003**
AST(GOT)	16(11:5)	53(38:15)	27.1 ± 4.4	26.5 ± 5.4	30.4 ± 4.7	29.2 ± 5.6	**< 0.001**	0.5378	0.4372
ALT(GPT)	15(12:3)	54(37:17)	24.6 ± 3.2	27.2 ± 5.4	27.6 ± 4.0	30.0 ± 5.6	**< 0.001**	0.0902	0.8594
ALP	15(9:6)	54(40:14)	27.3 ± 5.4	26.5 ± 5.1	30.0 ± 5.7	29.3 ± 5.3	**< 0.001**	0.6309	0.8943
LAP	16(12:4)	53(37:16)	24.8 ± 3.9	27.2 ± 5.4	27.9 ± 4.0	30.0 ± 5.7	**< 0.001**	0.1252	0.6677
LD(LDH)	17(10:7)	52(39:13)	28.7 ± 4.6	26.0 ± 5.1	31.3 ± 5.0	28.9 ± 5.4	**< 0.001**	0.068	0.5984
γ-GTP	14(13:1)	55(36:19)	23.8 ± 3.6	27.4 ± 5.2	26.2 ± 3.6	30.3 ± 5.5	**< 0.001**	**0.0105**	0.4903
TG	17(15:2)	52(34:18)	23.6 ± 3.3	27.6 ± 5.2	26.4 ± 4.3	30.5 ± 5.4	**< 0.001**	**0.0038**	0.9606
TC	17(9:8)	52(40:12)	27.4 ± 4.8	26.4 ± 5.3	29.3 ± 5.7	29.5 ± 5.3	**< 0.001**	0.7951	0.1247
HDL-C	17(9:8)	52(40:12)	28.7 ± 4.7	26.0 ± 5.1	32.0 ± 4.1	28.7 ± 5.5	**< 0.001**	**0.0326**	0.4299
LDL-C	17(9:8)	52(40:12)	27.2 ± 5.8	26.5 ± 4.9	28.7 ± 5.6	29.7 ± 5.3	**< 0.001**	0.9126	**0.0105**
HbA1c	15(10:5)	54(39:15)	28.1 ± 4.8	26.3 ± 5.2	31.1 ± 4.2	29.0 ± 5.6	**< 0.001**	0.1987	0.7482
FBS	16(13:3)	53(36:17)	27.0 ± 5.7	26.6 ± 5.0	30.7 ± 5.5	29.1 ± 5.3	**< 0.001**	0.4946	0.102

Data of muscle thickness are presented as mean ± SD

*WBC* white blood cell count, *RBC* red blood cell count, *Hb* hemoglobin, *Ht* hematocrit, *MCV* mean corpuscular volume, *MCH* mean corpuscular hemoglobin, *MCHC* mean corpuscular hemoglobin concentration, *PLT* platelet count, *TP* total protein, *Alb* albumin, *AST(GOT)* aspartate aminotransferase (glutamic oxaloacetic transaminase), *ALT(GPT)* alanine aminotransferase (glutamic pyruvic transaminase), *ALP* alkaline phosphatase, *LAP* leucine aminopeptidase, *LD(LDH)* lactate dehydrogenase, *γ-GTP* γ-glutamyltransferase, *TG* triglyceride, *TC* total cholesterol, *HDL-C* high-density lipoprotein cholesterol, *LDL-C* low-density lipoprotein cholesterol, *HbA1c* hemoglobin A1c, *FBS* fasting blood sugar

To verify the influence that serum Alb concentration and serum Alb levels (< 4.1 g/dL or not) had on the change in muscle thickness at the AT, binomial logistic regression analysis was performed with muscle thickness at the AT (increased as 0, decreased as 1) as a target variable and age, sex, serum Alb concentration, and serum Alb level (≥ 4.1 g/dL as 0, < 4.1 g/dL as 1) as explanatory variables. The serum Alb level was selected as a significant variable (*p* = 0.0102), and the odds ratio for decreasing muscle thickness at the AT was 7.08, while the 95 % confidence interval was 1.59–31.54.

## Discussion

This study revealed that a low-load resistance training program using a participant’s own body weight and elastic bands, even when performed only twice a week, induced muscle hypertrophy after 12 weeks of training intervention in community-dwelling elderly Japanese participants. The average muscle thickness at the AT increased by about 10 % over the 12 weeks of the low-load resistance training program. However, there were individual differences in the training effect on muscle thickness at the AT. For example, the increase was restricted in individuals with a relatively lower serum Alb before the training intervention. We first comprehensively verified the blood parameters that were included in the annual medical checkups conducted in Japan before and after the low-load resistance training program. Our data newly suggested that low serum Alb levels may predict decreased efficacy of a low-load resistance training intervention on muscle thickness in community-dwelling elderly Japanese participants. Hence, this may be a predictive biomarker for the effect of a low-load resistance training program on muscle hypertrophy.

It is common to divide participants using quartile values of blood parameters or based on physical performance tests [23, 24]; here, we compared the lowest quartile with the three higher quartiles combined as reported in a previous study [25]. When we divided the participants into two groups using each blood parameter’s quartile criteria (Table 1), an interaction between time and group was detected for MCHC, serum Alb levels, and serum LDL-C levels (Table 2). The training effect on muscle thickness at the AT was limited in the low serum Alb group compared with that in the normal serum Alb group (Fig. 2a). However, this result was neither confirmed in the low MCHC group compared with that in the normal MCHC group (Fig. 2b) nor in the low serum LDL-C group compared with that in the normal serum LDL-C group (Fig. 2c). From the results of the analysis of covariance, this was not affected by the participation rate of the classes. We also showed that the effect of low serum Alb level on muscle thickness at the AT was not affected by age and sex because these parameters were not selected as variables in the binomial logistic regression analysis. The results of the binomial logistic regression analysis showed that classification in the low serum Alb group had an odds ratio of 7.08 for decreasing muscle thickness at the AT, which supports the above-mentioned limitation. Although this criterion was much higher than that of clinical malnutrition (< 3.5 g/dL), the patients with relatively low serum Alb had inhibited training effects. This influence was not seen in participants with lower levels of the other blood parameters, including MCHC and LDL-C (Table 2; Fig. 2b, and Fig. 2c). Our data demonstrated that the effect of a low-load resistance training program on lower limb muscle thickness appears to be limited in participants, depending on their serum Alb levels before the training intervention. Serum Alb is a clinical indicator of energy and protein deficiency; therefore, our data suggest that participants with lower serum Alb before a training intervention ought to improve their nutritional status to obtain the most optimal training effects on their muscle mass. The relatively lower serum Alb level (3.9–4.2 g/dL) was also reported to be related to cognitive decline or dementia [26–28]. Some other previous longitudinal observational studies reported that relatively lower serum Alb levels were associated with loss of muscle mass and muscle strength [24, 29, 30]. It was also reported that a relatively lower serum Alb level combined with sarcopenia would increase disability risk in older adults [31]. As these studies and our data showed, even though the serum Alb level is much higher than that of clinical malnutrition (< 3.5 g/dL), relatively lower serum Alb level might be an indicator for our healthy aging. MCHC is one of the indexes for evaluating anemia, whereas serum LDL-C is one of the indexes for evaluating dyslipidemia. Although these two parameters might also reflect the nutritional status, only serum Alb had an effect of the low-load resistance training program on the lower limb muscle thickness. The relatively lower criteria that were not within clinically abnormal range, were similar for these three parameters. Therefore, serum Alb might detect a poor physical condition in the early stages.

Measuring the muscle thickness via a B-mode ultrasound device is a non-invasive method for assessing the muscle mass, which can also be assessed individually. The training effects tend to occur in site-specific manner; thus, this method is useful for assessing the training effects on muscle mass. Some RCTs in older adults revealed that exercise intervention improved muscle mass when assessed by dual energy X-ray absorptiometry [32] or segmental multifrequency bioelectrical impedance analysis [33]. Our data is important because it revealed that a low-load resistance training program is effective at increasing the site-specific muscle mass in community-dwelling middle-aged and older adults.

Our study had some limitations. First, we did not include a control group when we verified the effects of the 12-week training intervention, and we also did not conduct the reproducibility measures in these participants. Second, even though it has been reported that a combination of exercise and a nutritional approach is the most effective method to improve sarcopenia, we did not control for the participants’ nutrient intake during the training period. Third, although there were 71 % female participants included, we could not verify the sex difference when examining the association between serum Alb levels and the low-load resistance training effects. Fourth, the sample size of 69 was small. Fifth, although it was approved that all the participants join the exercise program by the physician in charge, their medical history or their use of oral medications might have affected the blood parameters, which we assessed in this study. To minimize these limitations, further studies must be conducted in the future.

## Conclusions

In conclusion, serum Alb levels may be a predictive biomarker for the effect of a low-load resistance training program on muscle hypertrophy in community-dwelling elderly individuals.

## Data Availability

The datasets analyzed during the current study are available from the corresponding author on reasonable request.

## Abbreviations

AT: The anterior aspects of the thigh
WBC: White blood cell count
RBC: Red blood cell count
Hb: Hemoglobin
Ht: Hematocrit
MCV: Mean corpuscular volume
MCH: Mean corpuscular hemoglobin
MCHC: Mean corpuscular hemoglobin concentration
PLT: Platelet count
TP: Total protein
Alb: Albumin
AST(GOT): Aspartate aminotransferase (glutamic oxaloacetic transaminase)
ALT(GPT): Alanine aminotransferase (glutamic pyruvic transaminase)
ALP: Alkaline phosphatase
LAP: Leucine aminopeptidase
LD(LDH): Lactate dehydrogenase
γ-GTP: γ-glutamyltransferase
TG: Triglyceride
TC: Total cholesterol
HDL-C: High-density lipoprotein cholesterol
LDL-C: Low-density lipoprotein cholesterol
HbA1c: Hemoglobin A1c
FBS: Fasting blood sugar
